# University freshmen's excessive smartphone use and psychological safety during the COVID-19 pandemic

**DOI:** 10.3389/fpsyt.2022.993555

**Published:** 2022-10-06

**Authors:** Qingsong Yang, Mengxi Shi, Lianping Zeng, Ping Xu

**Affiliations:** ^1^School of Teacher Education, Zunyi Normal University, Zunyi, China; ^2^School of Psychology, Guizhou Normal University, Guiyang, China; ^3^Department of Educational Psychology, Guangzhou Sport University, Guangzhou, China

**Keywords:** excessive smartphone use, psychological safety, hardiness, interpersonal distress, university freshmen

## Abstract

Although excessive smartphone use has been confirmed as being associated with specific representations of mental health (e. g., anxiety, depression, wellbeing, etc.) throughout the COVID-19 pandemic, the relationship between excessive smartphone use and cognitive representations of mental health (i.e., psychological safety) is not yet fully understood. This study aimed to identify the association between excessive smartphone use and psychological safety among university freshmen during the COVID-19 pandemic; in addition, we examined the mediation effects of hardiness and interpersonal distress in this relationship. In this study, 1,224 university freshmen were selected at random from several universities in Guizhou Province of China. The Psychological Safety Scale was used to evaluate the mental health of university freshmen; the Mobile Phone Dependence Scale was used to evaluate excessive smartphone use; the Hardiness Questionnaire was used to evaluate hardiness; and the Interpersonal Relation Synthetic Diagnose Test was used to evaluate interpersonal distress. The findings showed that: (1) the greater the degree of excessive smartphone use, the more serious respondents' interpersonal distress and the lower their hardiness; (2) excessive smartphone use was not only directly related to the psychological safety of university freshmen but also indirectly related to their psychological safety through the independent mediation of hardiness and interpersonal distress, as well as through the chain mediation of hardiness and interpersonal distress. In general, excessive smartphone use in university freshmen could lead to a decline in their psychological safety. Also, hardiness and interpersonal distress play a complex role in this relationship. During the COVID-19 pandemic, interventions on the mental health of college freshmen should not only provide guidance on how to use their smartphone responsibly but also to provide them with support and guidance for the enhancement of their hardiness and improvement of their interpersonal relationships.

## Introduction

For over 2 years, the entire world has struggled with the global COVID-19 pandemic. During this time, many households have faced isolation, fear, violence, drug abuse, and anxiety. The pandemic has affected not only physical health but also mental functioning (1). A recent study conducted among undergraduates in a Chinese college reported that 24.9% of the participants experienced psychological distress due to the COVID-19 pandemic (2). In another cross-sectional survey conducted in Iran, 99.0 and 69.6% of the participating medical students reported suffering from extremely severe anxiety and depression, respectively, due to commuting restrictions and fear of contracting the virus (3). These findings suggest that the pandemic has indeed had a negative impact on people's mental health.

To control the rapid spread of COVID-19, home quarantine and altered teaching styles for college students have been enforced in most colleges and universities. However, these policies may have led to an important unintended consequence, as time spent studying online may be a potential risk factor for excessive smartphone use, which could further result in negative psychological disorders. Relevant empirical findings have also provided support for this view. For example, a recent national survey of 746,217 Chinese college students showed that the risks of developing depression and anxiety disorders increased with their exposure time to smartphone use (4). Another study of 31,425 American college and graduate students also showed that problematic use of smartphones was demonstrably associated with certain mental health diagnoses (especially ADHD, anxiety, depression, and PTSD) (5).

Numerous related studies have been done on the relationship between excessive smartphone use and mental health (4, 5). However, the mechanism underlying this relationship during the COVID-19 pandemic has been inadequately explored. Moreover, previous research on the relationship between excessive smartphone use and mental health has focused on the analysis of representations (e.g., anxiety, depression, etc.) in mental health, but the analysis of cognitive representations (i.e., psychological safety) is still lacking. During the COVID-19 pandemic, psychological safety has been considered as an important indicator of mental health (6, 7). Therefore, the current study focused on the association between excessive smartphone use and psychological safety among college students during the COVID-19 pandemic. According to social cognitive theory, not only is people's behavior a response to the external environment and internal psychology (e.g., cognition, emotion, etc.), but also their behavior also has a direct effect on the external environment and their internal psychology (8). Based on this theory, the current study also intended to investigate the association mechanism of excessive smartphone use with psychological safety from both the external environment (interpersonal distress) and internal psychology (hardiness).

During the pandemic, many schools have shifted to online teaching for safety concerns. Smartphones have become an important tool for students when learning online (9). The selection of college freshmen for the current study was based on two considerations. First, college freshmen in general have recently been relieved of the pressure of the college entrance examination, and want to relax in college to compensate for their lack of time seeking entertainment in high school (10). Second, during the transition from high school to university, college freshmen generally face a variety of pressures including adapting to new environments, dealing with new interpersonal relationships, and embracing new ways of learning. These pressures often lead college freshmen to use their smartphones more to alleviate their burden of stress (11). These factors are likely to contribute to their excessive smartphone use. Therefore, it was determined that college freshmen would be a suitable demographic for this study.

### Excessive smartphones use and psychological safety

Excessive smartphone use (also referred to as smartphone addiction or smartphone dependence) refers to people's inability to control the amount of time they spend using their smartphones, leading to excessive smartphone use which negatively impacts their physical and mental health (12). According to media dependency theory, the more an individual relies on a particular medium to meet their needs, the greater the influence of that medium on them (13). Numerous studies have examined the relationship between excessive smartphone use and specific representations of mental health problems (e.g., anxiety, depression, etc.) during the COVID-19 pandemic, and have identified a positive relationship between smartphone use and poor mental health (4, 14). However, few studies have focused on the relationship between excessive smartphone use and psychological safety, and the mechanisms at play between them. Psychological safety can be used as a positive indicator reflecting mental health during the pandemic (6, 7).

Psychological safety is an internal need for stability, expressed primarily as a sense of certainty and control over people or things in one's environment. It is measured by a two-factor structure, composed of the interpersonal security and the certainty in control (15). According to Maslow's hierarchy of needs theory, psychological safety is one of the basic human needs, and the satisfaction of this need determines the healthy growth and development of an individual (16). On the behavioral level, psychological safety is mirrored by actively participating in social activity and developing peer relationships, positively exploring the external world, and exerting reasonable control over one's behavior in the face of setbacks and pressures (17). In fact, many human behaviors are designed to maintain a sense of psychological safety (15). Some researchers have considered psychological safety as an important factor in characterizing mental health (18). Others have even treated psychological safety and mental health as synonymous (6). There are two main sources of psychological safety: one is the perception of a safe environment; the other is the judgment of one's ability to cope with change (19). During the COVID-19 pandemic, while most people were in quarantine at home, many worked from or found entertainment through their smartphone (20). This led to more time and opportunity for people to use their smartphones. Excessive smartphone use will often cause individuals to experience loss of control (12), thus leading to them blocking sources of psychological safety. Therefore, as a starting point, the current study hypothesized that:

*Hypothesis* 1: Excessive smartphone use is negatively associated with psychological safety.

### The affecting mechanism of hardiness

In positive psychology, hardiness is seen as a set of personal resources that appear to protect individuals from the adverse effects of stress (21, 22), which is usually defined as a generalized style of functioning characterized by a high level of commitment, control, and challenge. Hardiness is considered by many to be a stable personality trait (23). However, some studies emphasize the variability in hardiness due to either extrinsic or intrinsic influences, and have regarded hardiness as a state trait, arguing that it shows not only relative stability similar to personality traits but also variability (21, 24). In fact, hardiness represents one's sustained effort and consistent focus on goal-pursuing processes, during which an individual will regulate their thoughts, emotions, and behaviors based on daily events or experiences (25), thus exhibiting variability during such a period of time. Relevant empirical findings have also provided support for this view. For example, Wong et al. found that hardiness changes dynamically due to daily self-concept clarity (25); Dymecka et al. showed that fear of COVID-19 would lead to the variability of hardiness among Polish people (26); Yu et al. also identified that students' mathematics anxiety weakened their hardiness in math learning (27). The current study therefore regarded hardiness as a state trait that can be affected by internal and external factors during a period of time.

Some consider hardiness to be a form of self-control in the face of stress or adversity (28). Studies have also found that hardiness is closely associated with brain regions related to self-control (29). Using resting-state functional magnetic resonance images (rs-fMRI), researchers have found that the more severe one's excessive smartphone use, the more pronounced the decline in the altered connectivity of an individual's right inferior frontal gyrus, and the poorer their self-control (30). According to the strength model of self-control, behaviors such as information processing, interpersonal interaction, and impression management all consume an individual's limited psychological resources. The depletion of psychological resources below a certain level leads to a failure in self-control (31). Browsing websites, socializing online, and self-presentation are all common smartphone usage behaviors. Therefore, excessive smartphone use can deplete an individual's limited mental resources, leading to a failure in their self-control (32), which in turn leads to a decrease in their level of hardiness. Therefore, the current study hypothesized the following:

*Hypothesis* 2: Excessive smartphone use is negatively associated with hardiness.

In the face of environmental changes or risks, hardy individuals have strong insight and self-control and believe that they are able to adapt to environmental changes, and that their abilities can be improved through their efforts to change unfavorable conditions or solve crises (21), thereby making their lives more fulfilling and safe. Empirical studies have shown that hardiness is negatively associated with loneliness and depressive symptoms, and that it has played a core role in protecting the mental health of individuals during the COVID-19 pandemic (33). That is, the higher one's level of hardiness, the higher their level of mental health; conversely, the lower one's level of hardiness, the more likely they are to suffer from mental health problems (34). Another empirical study has found a positive correlation between hardiness and psychological safety (21). As proposed by the Hypothesis 2, excessive smartphone use is likely related to hardiness, and as hardiness is shown to be closely related to psychological safety (21), we inferred that hardiness might play an indirect role in the relationship between excessive smartphone use and psychological safety. Therefore, the current study hypothesized the following:

*Hypothesis* 3: Hardiness is positively associated with psychological safety.

*Hypothesis* 4: Hardiness mediates the relationship between excessive smartphone use and psychological safety.

### The affecting mechanism of interpersonal distress

Interpersonal distress refers to the inability to communicate and interact with people normally, while exhibiting various symptoms including not being understood by others or appearing reserved and anxious when dealing with others (35). It can be divided into three types: interpersonal conversation distress, interpersonal communication distress, and heterosexual communication distress (36). College freshmen end or change their existing relationships when transitioning from high school to university, and are more likely to encounter interpersonal distress (10). Interpersonal distress has attracted research attention specifically as an important indicator of interpersonal environmental health (37). Excessive smartphone use has also been linked with interpersonal distress. Some studies have found that excessive smartphone use can easily lead to individuals ignoring interactions with people around them, thus negatively impacting interpersonal relationships (38, 39). Interpersonal relationships are often regarded as a reflection of an individual's abilities, and they affect all aspects of individual's life and learning (40). An individual who cannot handle interpersonal relationships well will face interpersonal distress as a result, which will likely become a serious stress on their mental health (41). Interpersonal distress destroys an individual's internal control and trust, making them feel their life is meaningless and affecting their mental health (42). According to the theory of emotional security, interpersonal distress induces adverse effects on an individual's psychological safety (43). Related studies have also found that good interpersonal relationships contribute to psychological safety (44). Therefore, the current study hypothesized the following:

*Hypothesis* 5: Excessive smartphone use is positively associated with interpersonal distress.

*Hypothesis* 6: Interpersonal distress is negatively associated with psychological safety.

*Hypothesis* 7: Interpersonal distress mediates the relationship between excessive smartphone use and psychological safety.

According to the hardiness model, hardiness is considered to be an important protective factor for mental health which reduces physical and psychological distress by attracting social support (22). In other words, hardy individuals generally have good interpersonal relationships. Empirical studies have also found that higher levels of hardiness are associated with lower levels of interpersonal distress (21). Therefore, the current study hypothesized the following:

*Hypothesis* 8: Hardiness is negatively associated with interpersonal distress.

*Hypothesis* 9: Hardiness and interpersonal distress play chain mediating roles between excessive smartphone use and psychological safety.

### The current study

**Figure 1:**
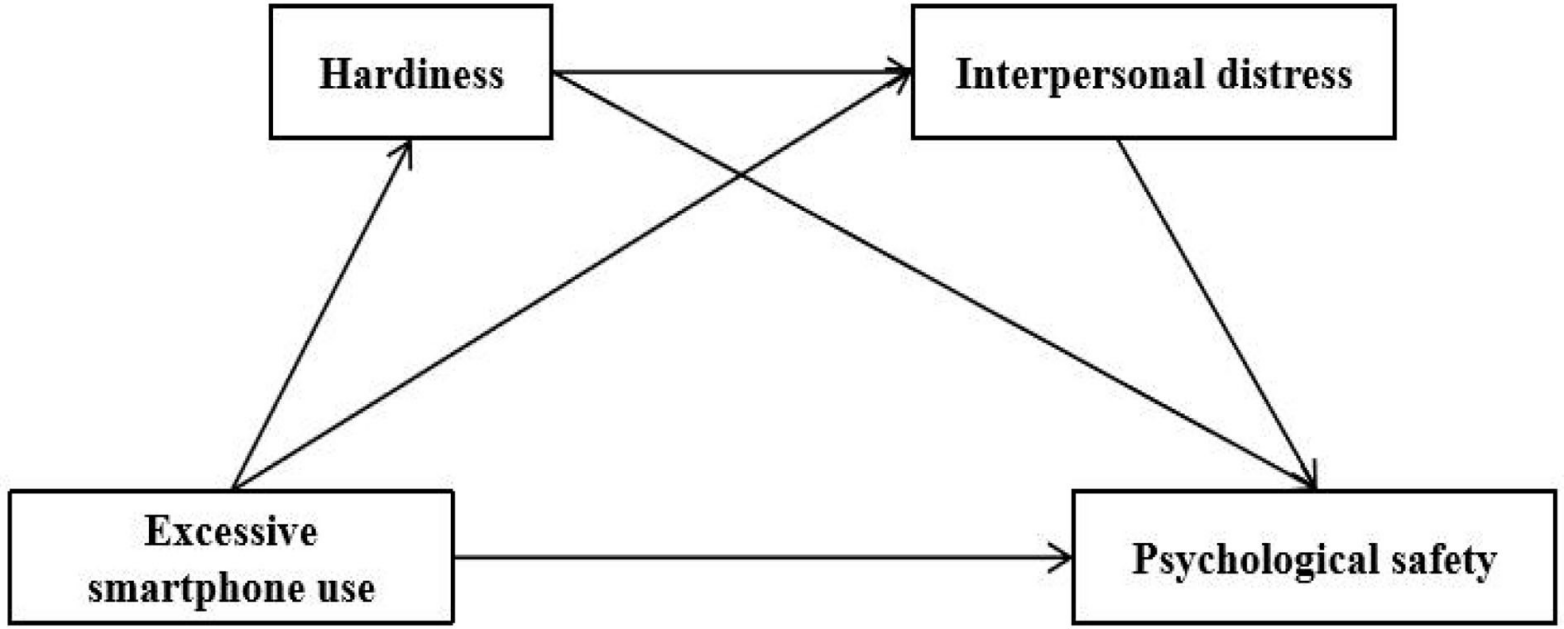
Proposed model of excessive smartphone use as a predictor of psychological safety mediated by hardiness and interpersonal distress.

To better understand the impact of excessive smartphone use on the mental health of university freshmen and the mechanisms at play during the COVID-19 pandemic, and guided by media dependence theory and social cognitive theory, the current study looked at university freshmen as the subject, and assessed their mental health using psychological safety as an indicator. The mechanisms were explored through the mediating effects of both internal psychology (hardiness) and the external environment (interpersonal distress). The hypothesized model is depicted in Figure 1. To ensure the reliability of the results, we controlled for variables that might influence the relationship between excessive smartphone use and psychological safety. Previous studies have found gender differences in excessive smartphone use (20), as well as differences relating to both gender and age with regard to hardiness (45). Therefore, we controlled for demographic variables including gender and age.

## Materials and methods

### Participants and procedure

The research group elected to use freshmen studying in Guizhou Province during September and October 2021 as the survey population. First, two universities, Guizhou Normal University and Zunyi Normal University, were selected at random from a list of 21 public universities in Guizhou Province. Then, 1,317 freshmen (out of a total of 10,022 freshmen at these two universities) were selected, also at random, as the survey subjects out of the 10,022 freshmen from Guizhou Normal University and Zunyi Normal University. A total of 93 incomplete or identical responses were removed. After data cleaning, 1,224 questionnaires were considered to be valid to use with an effective rate of 92.938%. The mean age of the subjects was 19.910 years old (*SD* = 0.873 years). Among these, female students accounted for 55.310% of the total. Detailed demographic data of the studied sample is presented in Table 1.

**Table 1:** Demographic information of the study sample (*N* = 1,224).

	***M***	***SD***	**Min**	**Max**
**Age**				
All participants	19.910	0.873	17	24
Female	20.060	0.673	19	24
Male	19.730	1.043	17	24
		**N**		**%**
**Sex**				
Male		677		44.690
Female		547		55.310
**Annual family income**				
<10,000 yuan		316		25.817
10,000–30,000 yuan		422		34.477
30,000–60,000 yuan		264		21.568
60,000–100,000 yuan		147		12.009
More than 100,000 yuan		75		6.127
**Number of siblings**				
Only child		121		9.885
Non-only child		1,103		90.115
**Residential location**				
Rural		1,100		89.869
Urban		124		10.131

During the investigation process, the researcher conducted group training for the members of the investigation team, ensuring standardized instruction procedures during the investigation. Before the survey, each investigation team member completed a questionnaire independently to familiarize themselves with the full questionnaire. All questions related to the survey were distributed to participants in a classroom setting using a paper form, and were collected anonymously on the spot. Investigators were present to respond to any problems encountered by participants during the study process. After the questionnaires were collected, invalid questionnaires were removed. The remaining valid questionnaires were encoded. Random checks were conducted after data entry to reduce entry errors. The study was approved by the Academic Committee of Zunyi Normal University, and written consent was obtained from each participant.

### Measures

#### Mobile phone dependence scale

The Mobile Phone Dependence Scale, edited by Huang (46), evaluates excessive smartphone use. The questionnaire comprises of 17 items in total measuring four dimensions: loss of control, withdrawal, avoidance, and ineffectiveness. Each item is rated on a five-point scale ranging from 1 (never) to 5 (always). The higher the total score, the more severe one's excessive smartphone use. It has been widely used and proven to have good validity and reliability for Chinese students (47). The internal consistency reliability coefficients in this study were 0.783 for the uncontrolled sub-scale, 0.859 for the withdrawal sub-scale, 0.751 for the avoidance sub-scale, 0.777 for the ineffectiveness sub-scale, and 0.901 for the total scale.

#### Hardiness questionnaire

The Hardiness Questionnaire, edited by Lu (48), evaluates hardiness. There are 27 items in total measuring the four dimensions: resilience, control, engagement, and challenge. Each item is rated on a four-point scale, with scoring options of “unsuitable”, “somewhat suitable”, “suitable”, to “very suitable”. The higher the one's total score, the stronger their personality hardiness. The reliability and validity of this questionnaire have been fully verified (49). The internal consistency reliability coefficients in this study were 0.757 for the resilience sub-scale, 0.834 for the control sub-scale, 0.805 for the engagement sub-scale, 0.791 for the challenge sub-scale, and 0.942 for the total scale.

#### Interpersonal relation synthetic diagnose test

The Interpersonal Relation Synthetic Diagnose Test evaluates interpersonal distress and was edited by Zheng (50). There are 28 items that measure a total of four dimensions: talking, socializing, dealing with people, and dating someone of the opposite sex. A score of 1 is given for “yes” and 0 for “no”. The higher the total score, the more serious one's interpersonal distress. The Chinese version of Interpersonal Relation Synthetic Diagnose Test has been confirmed to be reliable and valid among adolescents and adults (51). The internal consistency reliability coefficients in this study were 0.728 for the talking sub-scale, 0.779 for the socializing sub-scale, 0.648 for the dealing with people sub-scale, 0.711 for dating the opposite sex sub-scale, and 0.902 for the total scale.

#### Psychological safety questionnaire

The Psychological Safety Questionnaire was edited by Cong (15) and is used to evaluate psychological safety. There are 16 items in total measuring two dimensions: interpersonal security and certainty in control. Each item is rated on a five-point scale from 1 (“very suitable”) to 5 (“very unsuitable”). The higher the total score, the stronger one's psychological safety. It has been used widely and proven to have good validity and reliability for Chinese students (52). The internal consistency reliability coefficients in the current study were 0.877 for the interpersonal security sub-scale, 0.879 for the certainty in control sub-scale, and 0.933 for the total scale.

#### Covariates

In this study, the socioeconomic characteristics of the subjects were treated as covariates. This included age, sex (1 = male; 2 = female), annual family income (1 = <10,000 yuan, 2 = 10,000–30,000, 3 = 30,000–60,000 yuan, 4 = 60,000–100,000 yuan, 5 = more than 100,000 yuan), siblings (1 = only child, 2 = non-only child), and household registration (1 = rural, 2 = urban).

### Data analysis

A total of 93 incomplete or identical response results were removed. After data cleaning, 1,224 questionnaires were considered to be valid for use. Data analysis was performed using SPSS 26.0. First, we checked the bias degree of the common method in this study. Second, descriptive analysis and correlation analysis were adopted to test the mean and standard deviation of each variable and their correlation coefficients. Third, model six of SPSS macro PROCESS 3.5 (53) was used to test the mediation effect of hardiness and interpersonal distress in the relationship between interpersonal stress and psychological safety, and the significance of the mediation effect was further determined by generating the bias-corrected bootstrap confidence interval (using 5,000 bootstrapping samples). In addition, all variables, such as age, gender, family income, birth, and whether they were an only child, were treated as covariates.

## Results

### Common methodological bias

An unrotated exploratory factor analysis of all variables showed that there were 17 factors with characteristic roots >1. The amount of variation explained by the first factor was 18.323%, which did not exceed the critical criterion of 40%. Therefore, it can be concluded that there was no serious common methodological bias in the variables involved in this study.

### Descriptive and correlation analyses of each variable

As shown in Table 2, excessive smartphone use showed a positive correlation with interpersonal distress, as expected (*r* = 0.341, *p* < 0.001), and a negative correlation with hardiness (*r* = −0.101, *p* < 0.001) and psychological safety (*r* = −0.327, *p* < 0.001). Hardiness showed a negative correlation with interpersonal distress (*r* = −0.209, *p* < 0.001) and a positive correlation with psychological safety (*r* = 0.361, *p* < 0.001). Interpersonal distress showed a negative correlation with psychological safety (*r* = −0.615, *p* < 0.001).

**Table 2:** Mean, standard deviation, and correlation coefficient (*r*; 95% CI) of the main variables.

	**Mean**	***SD***	**1**	**2**	**3**	**4**
1. Excessive smartphone use	2.511	0.688	1			
2. Hardiness	2.445	0.461	−0.101*** (−0.164, −0.034)	1		
3. Interpersonal stress	0.297	0.221	0.341*** (0.283, 0.398)	−0.209*** (−0.265, −0.154)	1	
4. Psychological safety	3.253	0.771	−0.327*** (−0.388, −0.265)	0.361*** (0.303, 0.417)	−0.615*** (−0.652, −575)	1

N = 1,224; ***p <0.001.

### Mediating effect test

Table 3 shows that excessive smartphone use had a negative effect on hardiness (β = −0.091, *p* < 0.05). When excessive smartphone use and hardiness simultaneously predicted interpersonal distress, excessive smartphone use had a positive predictive effect on interpersonal distress (β = 0.314, *p* < 0.001), and hardiness had a negative effect on interpersonal distress (β = −0.173, *p* < 0.001). When excessive smartphone use, hardiness, and interpersonal distress simultaneously predicted psychological safety, excessive smartphone use had a negative effect on psychological safety (β = −0.125, *p* < 0.001), hardiness had a positive effect on psychological safety (β = 0.239, *p* < 0.001), and interpersonal distress had a negative effect on psychological safety (β = −0.523, *p* < 0.001).

**Table 3:** Regression analysis between variables.

**Regression Equation**		**Overall fit index**		**Significance of regression coefficients**		
**Outcome variable**	**Predictive variable**	***R***	***R^2^***	***F***	**β**	***t***
Hardiness		0.189	0.036	7.571***		
	Gender				−0.321	−5.445***
	Age				0.034	1.039
	Family annual income				−0.024	−0.986
	Only child				−0.023	−0.228
	Household registration				0.02	0.761
	Excessive smartphone use smartphone use smartphone use				−0.091	−3.181*
Interpersonal Distress		0.396	0.157	32.408***		
	Gender				0.052	0.936
	Age				0.022	0.703
	Family annual income				−0.064	−2.692**
	Only child				0.164	1.726
	Household registration				0.096	1.012
	Excessive smartphone use				0.314	11.792***
	Hardiness				−0.173	−6.463***
Psychological Safety		0.671	0.449	124.127***		
	Gender				−0.001	−0.033
	Age				0.036	1.433
	Family annual income				0.001	0.075
	Only child				0.001	0.008
	Household registration				0.069	0.901
	Excessive smartphone use				−0.125	−5.491***
	Hardiness				0.239	10.843***
	Interpersonal distress				−0.523	−22.548***

N = 1,224. *p < 0.05,**p < 0.01,***p < 0.001.

**Figure 2:**
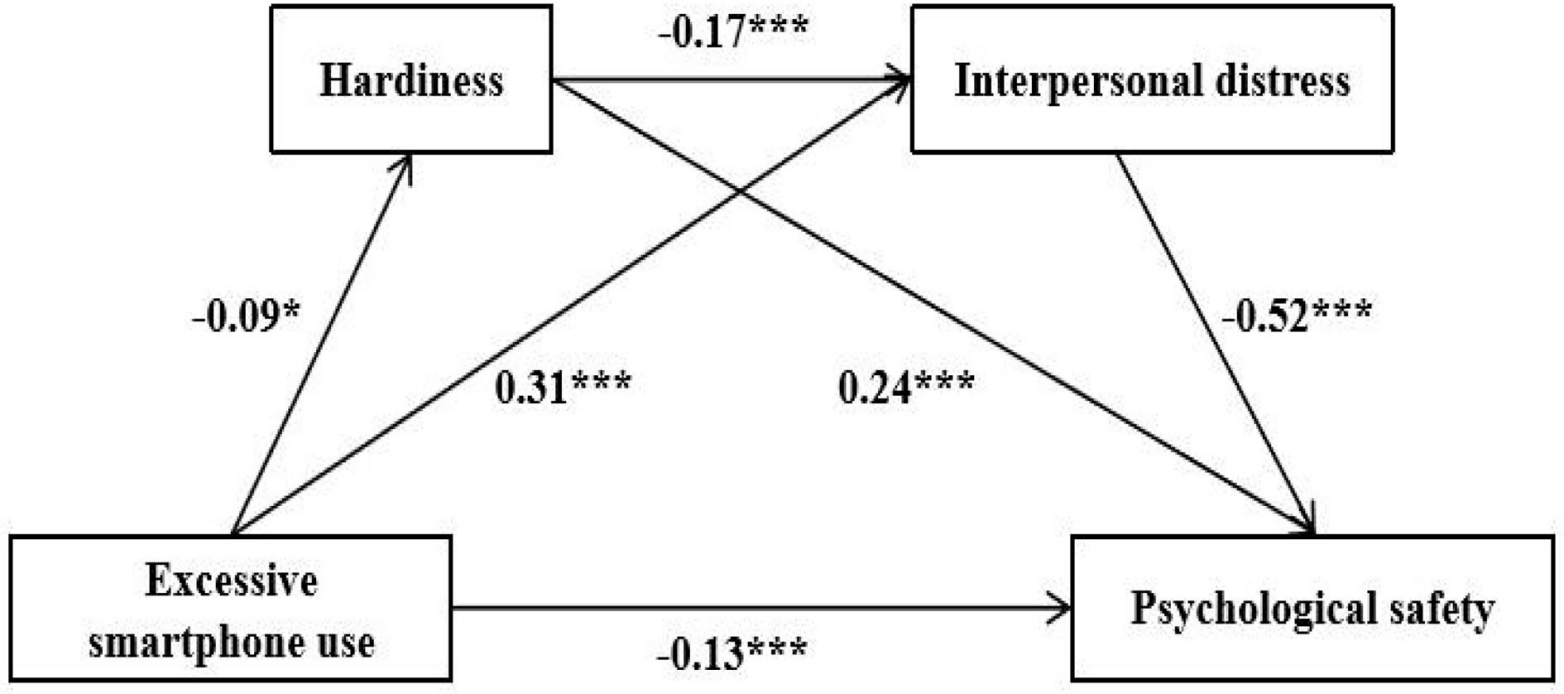
Mediation model of excessive smartphone use as a predictor of psychological safety mediated by hardiness and interpersonal distress. Standardized regression coefficients are displayed for all paths. *p < 0.05; ***p < 0.001.

Using the bias-corrected bootstrapped confidence intervals method, the sample was repeated 5,000 times at 95% confidence intervals (CI) to calculate the mediation effect. If 95% CI did not contain 0, it indicated a significant mediating effect. As shown in Table 4, the total mediating effect value was 0.193, accounting for 60.691% of the total effect of excessive smartphone use on psychological safety (0.318). First, we tested the mediating effect of hardiness between excessive smartphone use and psychological safety. It was found that the indirect effect of hardiness was −0.021 (95% CI = [−0.037, −0.006]), which indicates a significant mediating effect of hardiness, with the mediating effect accounting for 6.604% of the total effect. Second, we tested the mediating effect of interpersonal distress between excessive smartphone use and psychological safety. It was found that the indirect effect of interpersonal distress was −0.164 (95% CI = [−0.196, −0.131]), which indicated a significant mediating effect of interpersonal distress and the mediating effect accounted for 51.572% of the total effect. Third, we tested the chain mediating role of hardiness and interpersonal distress between excessive smartphone use and psychological safety. It was found that the indirect effect of hardiness and interpersonal distress was −0.008 (95% CI = [−0.015, −0.002]), which indicated a significant chain mediating effect of hardiness and interpersonal distress, with the mediating effect accounting for 2.515% of the total effect. The corresponding pathways are shown in Figure 2.

**Table 4:** Direct and mediating effects of excessive smartphone use on mental health.

**Effect**	**Path**	**Effect value**	**95% CI**	**Effect amount**
Total effect		−0.318	−0.001, −0.371	100%
Direct effect	Excessive smartphone use → Psychological safety	−0.125	−0.001, −0.169	39.308%
	Excessive smartphone use → Hardiness → Psychological safety	−0.021	−0.037, −0.006	6.604%
Mediating effect	Excessive smartphone use → Interpersonal distress → Psychological safety	−0.164	−0.196, −0.131	51.572%
	Excessive smartphone use → Hardiness → Interpersonal Distress → Psychological safety	−0.008	−0.015, −0.002	2.515%
Total mediating effect		−0.193	−0.231, −0.155	60.691%

## Discussion

Among the effects of the COVID-19 pandemic, people began to use their smartphones more often (20), and the influence of excessive smartphone use on mental health has received increasing attention from researchers. Previous studies have found that excessive smartphone use can lead to mental health issues such as anxiety, depression, and attention deficit. The current study extends our understandings of the effect of excessive smartphone use on mental health with regard to psychological safety, exploring the mechanisms of hardiness and interpersonal distress in the relationship between excessive smartphone use and psychological safety. All hypotheses were supported and some meaningful findings were revealed. First, excessive smartphone use was negatively associated with psychological safety. Second, both hardiness and interpersonal distress independently mediated the relationship between excessive smartphone use and psychological safety. Third, hardiness and interpersonal distress acted in a chain mediating role between excessive smartphone use and psychological safety. These findings provide further evidence for the theory of media dependence (13), while also pointing out the mechanism underlying the effect of excessive smartphone use on psychological safety in considering the mediating role of hardiness and interpersonal distress.

### Association of excessive smartphone use with psychological safety

This study found that excessive smartphone use was negatively associated with psychological safety. That is, the higher one's degree of excessive smartphone use, the lower their psychological safety. This finding supports the theory of media dependence in that the greater the degree of media dependence, the more severe its adverse effects (13). Previous studies have tended to analyze these effects with regard to negative emotions (54) or interpersonal distress (55). The present study focused on the cognitive dimension, i.e., psychological safety. Thus, this study expands our understanding of the effect of excessive smartphone use on mental health from the emotional and interpersonal levels to the cognitive level. We can understand the adverse effect of excessive smartphone use on psychological safety in this way: psychological safety is a manifestation of an individual's sense of control over themself and their environment (21), and the higher the degree of one's excessive smartphone use, the easier it is for them to lose control of themselves (12), thus resulting in a decline in their psychological safety. However, it is important to note that the problem of psychological safety in this study could not be attributed entirely to excessive smartphone use as we did not collect data regarding excessive smartphone use and psychological safety at a pre-pandemic timepoint. Some of these effects on mental health are likely to have arisen from the effects of the COVID-19 pandemic itself. Some longitudinal studies or meta-analysis studies comparing changes in mental health before and after the COVID-19 outbreak have found that the pandemic has induced more serious mental health problems (56, 57). Although these studies did not directly compare changes in psychological safety between the pre-pandemic period and during pandemic, they did provide inspiration for this study as the pandemic is likely to impair people's psychological safety. From this, we can speculate that in this study, part of the decline in psychological safety is likely due to the pandemic itself. Furthermore, we can also understand this phenomenon in this way: the special environment and conditions caused by the COVID-19 pandemic have given individuals the opportunity to use their smartphones excessively (20), which in turn has led to a decline in their psychological safety. Excessive use of smartphones is likely to be an indirect way that the COVID-19 pandemic has affected psychological safety, that is, as a mediator variable. However, this view does need to be further verified by further research.

### The mediating role of hardiness

Our findings show that excessive smartphone use was related to psychological safety through the mediation of hardiness. That is, excessive smartphone use can be related to psychological safety both directly and indirectly through hardiness. This finding supports the view of the relationship model between psychological quality and mental health (58), which describes psychological quality (e.g., hardiness) as an endogenous factor that determines one's level of individual mental health, with extrinsic risk factors playing a role in mental health through psychological quality. In the current study, this played out in two ways. First, excessive smartphone use was negatively associated with hardiness, which is consistent with previous findings that college students with more mobile phone addiction were less hardy (59). According to the strength model of self-control (31), individuals who overuse smartphone consume psychological resources (60) and, as these psychological resources are consumed, they become less likely to be able to actively engage in self-control and cope with stress and frustration, thus inducing cognitive failure (61), which then leads to a decrease in their level of hardiness. Second, the present study found that hardiness was positively associated with psychological safety. This is consistent with the findings of related studies that have found that hardiness was positively associated with the psychological safety of the elderly (62). This could be understood as hardiness, as a positive coping trait for individuals, provides the resources and conditions to solve difficulties and resolve stress in stressful situations, and facilitates individual self-efficacy (63). Hardy individuals are more likely to pursue meaning and purpose in life (64), and are more inclined to believe that their capabilities can be enhanced through their own efforts (65). Moreover, hardy individuals are more likely to receive social support (62), resulting in a more fulfilling and secure life. The present study found that excessive smartphone use is related to psychological safety through the mediation of hardiness. To some extent, these findings enrich and extend media dependence theory, that is, that greater media dependence will deplete an individual's positive psychological resources (e.g., hardiness) and have adverse effects on psychological health.

### The mediating role of interpersonal distress

The present study found that interpersonal distress mediated the relationship between excessive smartphone use and psychological safety in two ways. First, excessive smartphone use was positively associated with interpersonal distress, which supports the theory of media dependence (13). This result is also consistent with the empirical results that mobile phone addiction can lead to interpersonal distress in college students from Guangxi Province of China (39). According to social displacement theory, individuals who spend a lot of time and energy in the virtual world lack realistic opportunities to engage and interact with others in person (66), thus resulting in poorer interpersonal relationships. Second, interpersonal distress was negatively associated with psychological safety. This result supports the theory of emotional security, which states that poor relationships tend to impair psychological safety (43). Moreover, the results of this study are also consistent with related research findings which have found that the lower the degree of interpersonal distress in the elderly, the higher their psychological safety (44). According to self-determination theory, the satisfaction of the need for belonging is a basic prerequisite for one's healthy growth and development (67). If an individual encounters interpersonal distress, such as being rejected or neglected, their sense of belonging cannot be satisfied. This sense of belonging is very important for Chinese people in particular who are collectivist-value-oriented (68), meaning that a lack of a sense of belonging will lead to a decline in their level of psychological safety.

The current study found that excessive smartphone use was associated with psychological safety through the mediation of interpersonal distress. The findings of this study enrich and extend media dependence theory in that media dependence can disrupt the real interpersonal environment (i.e., cause interpersonal distress) and have adverse effects on one's mental health. It is worth noting that, in this study, we included interpersonal distress as a consequence variable of excessive smartphone use. However, some studies have viewed interpersonal distress as an antecedent variable influencing excessive smartphone use (69). Causal inferences between interpersonal distress and excessive smartphone use cannot be drawn in this study. We hypothesize that there could be a bidirectional relationship between excessive smartphone use and interpersonal distress (i.e., excessive smartphone use affects interpersonal distress while interpersonal distress also affects excessive smartphone use). Future research should further explore this relationship from the perspective of bidirectional causality through longitudinal design.

### The chain mediating role of hardiness and interpersonal distress

The present study found that hardiness and interpersonal distress mediated the relationship between excessive smartphone use and psychological safety. That is, excessive smartphone use was associated with interpersonal distress through hardiness, which led to a decrease in psychological safety. This result indicates that there is a close relationship between hardiness and interpersonal distress, which supports the view of the hardiness model. This is also consistent with previous findings that an adverse parent–child relationship is negatively related to predicting the level of hardiness in college students (70). This can be interpreted as individuals with a high level of hardiness have more positive perceptions of interpersonal relationships (71) and, as such are more likely to tolerate others' faults and respond positively to interpersonal conflicts and problems, thus reducing interpersonal distress and improving interpersonal relationships (72). In contrast, individuals with a low level of hardiness are more intolerant of others' faults and are prone to having conflict with others, thus leading to interpersonal troubles.

Overall, the association of excessive smartphone use with psychological safety likely has multiple pathways. Excessive smartphone use could be directly related to psychological safety, or it could be indirectly related through the independent mediating effects of hardiness or interpersonal distress, or even through the chain mediating effect of hardiness and interpersonal distress. These findings help further our understanding of the mechanism of excessive smartphone use on psychological safety and provide insight into how to improve people's psychological safety particularly during the COVID-19 pandemic. We can consider interventions from three aspects: smartphone use, hardiness, and interpersonal distress. In terms of smartphone use, this can be reduced by restricting smartphone usage time and increasing activities with family members or peers. In terms of hardiness, this can be increased by improving coping styles and focusing on increasing university freshmen's motivation (73). As for interpersonal distress, this can be decreased by teaching university freshmen interpersonal skills and organizing group activities to build their interpersonal communication skills and connection. It should be noted that the mediating effect analysis found that the effect sizes of the independent mediating effect of hardiness and the chain mediating effect of hardiness and interpersonal distress between excessive smartphone use and psychological safety were only 6.60 and 2.52%, respectively, which are insignificant. Meanwhile, the effect sizes of the direct effect of excessive smartphone use and the independent mediating effect of interpersonal distress were 39.31 and 51.57%, respectively. In other words, the effect of excessive smartphone use on psychological safety was shown to work primarily through direct and indirect paths through interpersonal distress. This suggests that future interventions for psychological safety should focus on limiting smartphone usage time and improving interpersonal relationships.

### Limitations of the study

Although valuable findings have been revealed in this study, some limitations cannot be denied. First, this study investigated the association of excessive smartphone use with psychological safety and its mechanism with cross-sectional design during the COVID-19 pandemic, so only inter-variate correlations can be described and no causal inferences can be made. The stresses induced by the COVID-19 pandemic can also have affected psychological safety (74). Because of not being able to access pre-pandemic data, we were unable to separate the effects of excessive smartphone use on psychological safety from that of the COVID-19 pandemic itself. In other words, the effect of psychological safety in this study is likely to be driven by both excessive smartphone use and the COVID-19 pandemic. Moreover, some studies have suggested that there may be reciprocal causation between excessive smartphone use and mental health in that excessive smartphone use affects mental health while mental health status also affects excessive smartphone use (75). Longitudinal and experimental studies are needed to further explore the association of excessive smartphone use with psychological safety. Second, this study explored the association of excessive smartphone use with psychological safety and its mechanism, and the total effect was 0.318, which is not a high value. This implies that other factors have also affected psychological safety during the COVID-19 pandemic that were not addressed in this study, such as one's degree of exposure to the COVID-19 pandemic circumstances, their available coping resources, and present conditions, all of which have been found to be important factors affecting psychological safety during the COVID-19 pandemic (76). Future studies should further explore the influence of these factors and their mechanisms. Third, this study was conducted among university freshmen and did not include other groups as university freshmen were identified as having special characteristics in interpersonal settings (10). Whether the results of this study can be generalized to other groups requires further study.

## Conclusion

This study focused on the association of university freshmen's excessive smartphone use with psychological safety and its mechanism in the context of the COVID-19 pandemic. The findings confirmed that there are multiple pathways in the association. Specifically, excessive smartphone use could be directly related to psychological safety. Meanwhile, hardiness, interpersonal distress, and the combination of hardiness and interpersonal distress together also mediated the relationship between excessive smartphone use and psychological safety. The effect of excessive smartphone use on psychological safety appears to work through both direct and indirect paths through interpersonal distress. Future attention should focus on how to control smartphone use and improve interpersonal relationships in order to improve the psychological safety of university freshmen.

## Data availability statement

The original contributions presented in the study are included in the article/supplementary materials, further inquiries can be directed to the corresponding author.

## Ethics statement

The studies involving human participants were reviewed and approved by Review Committee, School of Teacher Education, Zunyi Normal University. The patients/participants provided their written informed consent to participate in this study.

## Author contributions

QY conceived and designed the study. MS and PX contributed to data collection. LZ analyzed the data. QY, MS, and PX wrote the paper. All authors reviewed and approved the manuscript.

## Funding

This research was supported by 2021 Guangdong Province Key Scientific Research Platform and Project (2021ZDZX4070, 2021GXJK619, and SPRITS202101) and 2022 Educational Science Planning Project in Guizhou Province (2022B072).

## Conflict of interest

The authors declare that the research was conducted in the absence of any commercial or financial relationships that could be construed as a potential conflict of interest.

## Publisher's note

All claims expressed in this article are solely those of the authors and do not necessarily represent those of their affiliated organizations, or those of the publisher, the editors and the reviewers. Any product that may be evaluated in this article, or claim that may be made by its manufacturer, is not guaranteed or endorsed by the publisher.
